# Desigualdades en salud según régimen de afiliación y eventos notificados al Sistema de Vigilancia (Sivigila) en Colombia, 2015

**DOI:** 10.7705/biomedica.4453

**Published:** 2019-12-30

**Authors:** Liliana Hilarión-Gaitán, Diana Díaz-Jiménez, Karol Cotes-Cantillo, Carlos Castañeda-Orjuela

**Affiliations:** 1 Observatorio Nacional de Salud, Instituto Nacional de Salud, Bogotá, D.C., Colombia Observatorio Nacional de Salud Instituto Nacional de Salud BogotáD.C Colombia

**Keywords:** disparidades en el estado de salud, sistemas de salud, vigilancia en salud pública, factores socioeconómicos, Colombia, health status disparities, health systems, public health surveillance, socioeconomic factors, Colombia

## Abstract

**Introducción.:**

Las desigualdades en salud se generan por diferencias en las condiciones sociales y económicas, lo cual influye en el riesgo de enfermar y la forma de enfrentar la enfermedad.

**Objetivo.:**

Evaluar las desigualdades sociales en salud en Colombia, utilizando el tipo de afiliación al sistema de salud como un parámetro representativo (proxy) de la condición socioeconómica.

**Materiales y métodos.:**

Se trata de un análisis descriptivo y retrospectivo en el que se calcularon las tasas específicas de incidencia, ajustadas por edad y sexo, para eventos de notificación obligatoria, utilizando el régimen de afiliación (subsidiado o contributivo) como variable representativa del nivel socioeconómico. Las estimaciones se hicieron a nivel departamental para el 2015. Las desigualdades sociales se calcularon en términos de brechas absolutas y relativas.

**Resultados.:**

Se evidencian desigualdades sociales en la ocurrencia de eventos de notificación obligatoria, las cuales desfavorecen a la población afiliada al régimen subsidiado. En esta población, se reportaron 82,31 casos más de malaria *Plasmodium falciparum* por 100.000 afiliados, que los notificados en el régimen contributivo. Respecto a la brecha relativa, el pertenecer al régimen subsidiado se asocia con un aumento de 31,74 veces del riesgo de morir por desnutrición en menores de cinco años. Otros eventos también presentaron profundas desigualdades, como los relacionados con la salud sexual y reproductiva (mortalidad materna, sífilis gestacional y sífilis congénita), las enfermedades infecciosas y las enfermedades transmisibles relacionadas con la pobreza (lepra y tuberculosis).

**Conclusión.:**

El tipo de afiliación al *Sistema General de Seguridad Social en Salud* en Colombia es un buen indicador del nivel socioeconómico, y es un factor predictor de mayor morbilidad y mortalidad prematura asociada con los factores determinantes sociales de la salud.

## Abstract (en)

**Introduction:**

Inequalities in the health field are caused by the differences in the social and economic conditions, that influence the disease risk and the measures taken to treat the disease.

**Objective:**

We aimed to estimate the social inequalities in health in Colombia, according to the type of affiliation to the health system as a proxy of socioeconomic status.

**Materials and methods:**

We conducted a retrospective descriptive analysis calculating incidence rates age and sex adjusted for all mandatory reporting events using the affiliation regime (subsidized and contributory) as a socioeconomic proxy. Estimates were made at departmental level for 2015. Social inequalities were calculated in terms of absolute and relative gaps.

**Results:**

We found social inequalities in the occurrence of mandatory reporting events in population affiliated to the Colombian subsidized regime (poor population). In this population, 82.31 cases of *Plasmodium falciparum* malaria per 100,000 affiliates were reported more than those reported in the contributory regime. Regarding the relative gap, belonging to the subsidized regime increased by 31.74 times the risk of dying from malnutrition in children under 5 years of age. Other events such as those related to sexual and reproductive health (maternal mortality, gestational syphilis and congenital syphilis); neglected diseases and communicable diseases related to poverty (leprosy and tuberculosis), also showed profound inequalities.

**Conclusion:**

In Colombia there are inequalities by regime of affiliation to the health system. Measured socioeconomic status was a predictor of increased morbidity and premature mortality.

Las inequidades en salud son innecesarias, evitables e injustas, y se basan en las barreras que impiden que las personas obtengan condiciones de vida favorables ^1^. Las desigualdades en salud se generan por diferencias en las condiciones sociales y económicas, que influyen en los comportamientos de la población, sus estilos de vida, el riesgo de enfermar y las medidas adoptadas para hacer frente a la enfermedad ^2^^,^^3^. Estas desigualdades sociales deben ser identificadas, hay que estudiar sus causas, conocerlas, hacerles seguimiento y explicarlas mediante un sistema de vigilancia de la salud poblacional, pues su medición y caracterización permiten actuar sobre los determinantes sociales de la salud ^3^.

En Colombia persisten dichas desigualdades en salud. En las poblaciones con más necesidades básicas insatisfechas (NBI), se han encontrado las tasas más altas de desnutrición, bajo peso al nacer, mortalidad por enfermedad diarreica aguda y mortalidad por infección respiratoria aguda ^4^. En ocasiones, el evidenciar la existencia de desigualdades sociales es complejo; pese a los avances en los sistemas de información de salud en el país, aún persisten limitaciones.

El sistema de salud colombiano se ha caracterizado por no contar con datos públicos completos, ni con registros adecuados y unificados, que permitan obtener información actualizada sobre el estado de salud de la población de forma rápida y sencilla, lo cual ha llevado a la segmentación del mismo y a problemas en el registro de la calidad de la atención ^5^^,^^6^. Esta situación ha venido mejorando bajo el liderazgo del Ministerio de Salud y Protección Social, que ha creado y dispuesto un Sistema de Información en Salud para el análisis de los datos oficiales del país ^7^.

El sistema de salud colombiano tiene diversas modalidades de afiliación; los dos regímenes que cuentan con mayor población afiliada son el contributivo y el subsidiado. Al régimen contributivo, pertenecen personas con capacidad de pago, vinculadas mediante un contrato de trabajo, servidores públicos, pensionados y trabajadores independientes. Las personas pertenecientes al régimen subsidiado, por lo general, son de escasos recursos y están clasificadas en los niveles 1 o 2 del Sisbén (Sistema de Selección de Beneficiarios para programas sociales); son poblaciones especiales prioritarias, personas en condición de desplazamiento, menores desvinculados del conflicto armado, comunidades indígenas, personas mayores en centros de protección, población rural migratoria, personas del programa de protección a testigos, indigentes o población gitana ^8^. En esta medida, estos dos regímenes de afiliación representan poblaciones con condiciones socioeconómicas diferentes: los del régimen contributivo tienen ‘mejores’ condiciones que los del régimen subsidiado.

El objetivo del presente análisis fue avanzar en la medición de las desigualdades sociales en salud en Colombia, por medio de la combinación de fuentes de información oficiales, a partir del análisis de los eventos de interés de notificación obligatoria en salud pública, utilizando los niveles de afiliación contributivo y subsidiado como un indicador de la condición socioeconómica de los pacientes reportados al sistema de vigilancia.

## Materiales y métodos

Este es un análisis descriptivo y retrospectivo de los eventos de interés en salud pública en Colombia, reportados al Sistema de Vigilancia en Salud Pública (Sivigila), según el régimen de afiliación en salud para el 2015. Cada evento fue analizado de acuerdo con la afiliación al Sistema Seguridad Social en Salud, cuyo objetivo es regular el servicio público esencial de salud y crear condiciones de acceso para toda la población residente del país. Sus modalidades de afiliación son la contributiva (capacidad de pago), la subsidiada (sin capacidad de pago), la especial y de excepción (magisterio y fuerzas militares), la no asegurada y aquella sin información.

Se calcularon las tasas de incidencia y de mortalidad nacionales y departamentales para el régimen de afiliación contributivo y subsidiado, ajustadas por edad y sexo; se calcularon por cada 100.000 personas mediante un método directo, utilizando la población de referencia de la Organización Mundial de la Salud (OMS) 2001 ^9^. Los numeradores correspondieron al número de casos reportados al Sivigila para cada evento incluido, mientras que los denominadores poblacionales se construyeron a partir de la base de datos única de afiliados del Fondo de Solidaridad y Garantía (Fosyga) ^10^, de la cual se extrajo el reporte del número de afiliados, según edad, sexo y tipo de régimen, con corte al mes de diciembre de 2015. Los demás regímenes reportaron valores muy bajos de población afiliada (menos del 3,0 %) y, por lo tanto, no se consideraron.

Se calcularon las medidas de brecha absoluta y relativa para el análisis de las desigualdades sociales, como la diferencia y la razón entre las tasas de incidencia para el régimen subsidiado y el contributivo, respectivamente, para cada evento y departamento, evaluando las brechas que desfavorecen a la población afiliada al régimen subsidiado. Debido a la cantidad de información obtenida, en la primera parte de los resultados se hace una breve descripción del número de casos notificados al sistema de vigilancia según la categoría de afiliación (contributivo, subsidiado, especial, excepción, sin información y no asegurado), mencionando los cinco eventos más frecuentes.

En la segunda parte, se presentan los resultados de las tasas nacionales ajustadas por edad y sexo para los 61 eventos reportados, junto con sus respectivas mediciones de desigualdad según el régimen de afiliación. Finalmente, se describen los resultados de las tasas departamentales ajustadas por edad y sexo. Todos los análisis se realizaron en el lenguaje de programación R, versión 3.3.1, y Microsoft Excel.

### Consideraciones éticas

Este estudio cumple con las normas científicas establecidas en la Resolución 008430 de 1993.

## Resultados

Se notificaron 630.598 casos al Sivigila durante 2015, de los cuales, 296.529 (47,02 %) correspondieron a afiliados al régimen subsidiado, 265.904 (42,17 %) al contributivo, 15.577 (2,47 %) a los regímenes especiales, y 15.488 (2,46 %) a los regímenes de excepción; 36.600 (5,80 %) se notificaron como personas no aseguradas y sobre 500 (0,07%) personas no se tenía información de afiliación al Sistema General de Seguridad Social en Salud.

Los cinco eventos más reportados a nivel nacional durante el 2015 fueron: en el régimen contributivo, varicela (25,13 %), agresiones por animales potencialmente transmisores de rabia (18,47 %), dengue (17,57 %), violencia de género (sic) (8,48 %) y Chikungunya (8,10 %); en el régimen subsidiado, agresiones por animales potencialmente transmisores de rabia (16,23 %), violencia de género (sic) (14,53 %), dengue (13,30 %), varicela (12,07 %) y malaria por *Plasmodium vivax* (6,97 %); en el régimen especial, agresiones por animales potencialmente transmisores de rabia (18,83 %), varicela (18,00 %), dengue (14,30 %), Chikungunya (11,87 %) y violencia de género (sic) (6,51 %); en el régimen de excepción, leishmaniasis cutánea (23,70 %), dengue (21,30 %), varicela (20,24 %), agresiones por animales potencialmente transmisores de rabia (11,77 %) y violencia de género (sic) (4,43 %); y en población no asegurada, malaria por *Plasmodium falciparum* (16,20 %), malaria por *P. vivax* (14,73 %), violencia de género (sic) (13,40 %), agresiones por animales potencialmente transmisores de rabia (13,78 %) y dengue (8,73 %).

De los 61 eventos notificados al Sivigila durante el 2015 y analizados en el presente artículo, 37 presentaron tasas de incidencia o de mortalidad más altas en el régimen subsidiado. Especialmente, para aquellos eventos trazadores de la calidad de la atención en salud, como: mortalidad en menores de cinco años por infección respiratoria aguda, enfermedad diarreica aguda y desnutrición; eventos relacionados con salud sexual y reproductiva, como mortalidad materna, sífilis gestacional y sífilis congénita; enfermedades infecciosas, como leishmaniasis, enfermedad de Chagas y malaria; y enfermedades transmisibles relacionadas con la pobreza, como lepra y tuberculosis (cuadro 1).

**Cuadro 1:** Tasas de incidencia y mortalidad (ajustadas por edad y sexo) por 100.000 personas para el régimen contributivo y el subsidiado, y desigualdades entre regímenes para eventos de notificación obligatoria, Colombia, 2015

Evento	Contributivo	Subsidiado†	Desigualdad absoluta‡			Desigualdad relativa§		
				(IC_95_%)			(IC_95_%)	
					
Violencia de género (sic)	117.75	189,25	71,5	(69,21 a	73,79)	1,61	(1,58 a	1,63)
Agresiones por animales potencialmente transmisores de rabia	231,36	182,57	-48,79 (-51,46 a -46,12)			0,79	(0,78 a	0,8)
Dengue	229,72	161,93	-67,79 (-70,39 a -65,18)			0,7	(0,7 a	0,71)
Varicela	336,32	138,3	-198,02 (-00,9 a -195,14)			0,41	(0,41 a	0,42)
Malaria por *Plasmodium falciparum*	7,04	89,35	82,31	(81,04 a	83,57)	12,69	(12,04 a	13,37)
Chikungunya	101,43	80,13	-21,3	(-23,07 a -19,53)			0,79	(0,77 a	0,81)
Malaria por *Plasmodium vivax*	6,81	77,88	71,07	(69,88 a	72,26)	11,43 (10,84 a 12,06)		
Bajo peso al nacer	46,75	46,3	-0,45	(-1,72 a	0,82)	0,99	(0,96 a	1,02)
Morbilidad materna extrema	42,31	39,6	-2,71	(-3,9 a	-1,52)	0,94	(0,91 a	0,96)
Mortalidad perinatal y neonatal	20,28	30,14	9,86	(8,93 a	10,79)	1,49	(1,43 a	1,54)
Intoxicación por plaguicidas	12,23	28,56	16,33	(15,5 a	17,16)	2,34	(2,23 a	2,44)
Tuberculosis pulmonar	13,47	27,71	14,24	(13,4 a	15,07)	2,06	(1,97 a	2,15)
HIV/sida - mortalidad por sida	27,75	26,78	-0,97	(-1,94 a	0)	0,97	(0,93 a	1)
Intoxicación por medicamentos	31,97	21,30	-10,66 (-11,63 a		-9,7)	0,67	(0,64 a	0,69)
Leishmaniasis cutánea	2,19	18,50	16,31	(15,72 a	16,9)	8,44	(7,68 a	9,29)
Defectos congénitos	15,42	16,55	1,13	(0,38 a	1,87)	1,07	(1,02 a	1,12)
Intoxicación por sustancias psicoactivas	19,32	15,51	-3,81	(-4,59 a	-3,03)	0,8	(0,77 a	0,84)
Accidente ofídico	2,23	14,83	12,6	(12,07 a	13,14)	6,65	(6,04 a	7,31)
Sífilis gestacional	6,13	14,63	8,5	(7,9 a	9,09)	2,39	(2,24 a	2,54)
Intoxicación por otras sustancias químicas	9,72	9,85	0,13	(-0,45 a	0,71)	1,01	(0,96 a	1,08)
Parotiditis	19,76	6,56	-13,2	(-13,88 a -12,52)			0,33	(0,31 a	0,35)
Tuberculosis extrapulmonar	4,87	4,78	-0,09	(-0,5 a	0,32)	0,98	(0,9 a	1,07)
Lesiones por artefactos explosivos	3,26	4,42	1,16	(0,8 a	1,52)	1,36	(1,23 a	1,49)
Hepatitis B y delta	4,49	4,25	-0,24	(-0,62 a	0,15)	0,95	(0,87 a	1,04)
Dengue grave	2,54	2,94	0,4	(0,09 a	0,71)	1,16	(1,03 a	1,3)
Sífilis congénita	0,57	2,83	2,26	(2,02 a	2,5)	4,98	(4,1 a	6,04)
Malaria asociada (formas mixtas)	0,20	2,42	2,22	(2,01 a	2,43)	12,3	(9 a	16,82)
Tosferina	2,53	2,37	-0,16	(-0,45 a	0,13)	0,94	(0,83 a	1,05)
Malaria complicada	0,72	2,09	1,37	(1,15 a	1,59)	2,9	(2,42 a	3,48)
Hepatitis A	3,56	1,92	-1,64	(-1,95 a	-1,33)	0,54	(0,48 a	0,61)
Intoxicación por solventes	1,96	1,87	-0,09	(-0,34 a	0,17)	0,96	(0,84 a	1,09)
Mortalidad materna	0,83	1,85	1,02	(0,81 a	1,24)	2,24	(1,88 a	2,67)
Mortalidad por IRA en menores de cinco años	0,73	1,78	1,05	(0,84 a	1,26)	2,43	(2,02 a	2,92)
Leptospirosis	1,43	1,74	0,31	(0,08 a	0,54)	1,22	(1,05 a	1,41)
Enfermedad de Chagas	0,58	1,46	0,88	(0,69 a	1,07)	2,52	(2,05 a	3,09)
ESAVI	1,72	1,22	-0,5	(-0,72 a	-0,27)	0,71	(0,61 a	0,83)
Cáncer infantil	2,25	1,18	-1,07	(-1,32 a	-0,83)	0,52	(0,45 a	0,61)
Tuberculosis farmacorresistente	0,51	1,03	0,52	(0,36 a	0,69)	2,03	(1,62 a	2,54)
Lepra	0,47	0,98	0,51	(0,36 a	0,67)	2,1	(1,66 a	2,66)
Intoxicación por gases	2,50	0,96	-1,53	(-1,78 a	-1,29)	0,39	(0,33 a	0,45)
Mortalidad por desnutrición en menores de cinco años	0,03	0,89	0,86	(0,74 a	0,99)	31,74	(14,12 a	71,35)
Leucemia linfoide aguda pediátrica	1,25	0,88	-0,37	(-0,56 a	-0,18)	0,7	(0,59 a	0,84)
Intoxicaciones por metales pesados	0,43	0,81	0,38	(0,23 a	0,52)	1,87	(1,46 a	2,4)
Intoxicación por metanol	0,44	0,59	0,14	(0,01 a	0,28)	1,33	(1,02 a	1,72)
Fiebre tifoidea y paratifoidea	0,58	0,46	-0,12	(-0,25 a	0,02)	0,79	(0,61 a	1,03)
Mortalidad por EDA en menores de cinco años	0,04	0,43	0,39	(0,3 a	0,48)	11,81	(5,68 a 24,55)	
EDA por rotavirus	0,06	0,39	0,33	(0,24 a	0,42)	6,32	(3,55 a	11,26)
Letalidad por dengue	0,22	0,34	0,13	(0,03 a	0,23)	1,59	(1,11 a	2,28)
Hepatitis C	0,50	0,3	-0,2	(-0,32 a	-0,08)	0,6	(0,44 a	0,81)
Meningitis por neumococo	0,25	0,25	0	(-0,09 a	0,09)	1	(0,69 a	1,45)
Leucemia mieloide aguda pediátrica	0,24	0,18	-0,06	(-0,15 a	0,02)	0,74	(0,5 a	1,12)
Leishmaniasis mucosa	0,04	0,14	0,11	(0,05 a	0,16)	3,89	(1,79 a	8,48)
Tétanos accidental	0,03	0,13	0,1	(0,05 a	0,15)	4,4	(1,86 a	10,38)
Meningitis	0,09	0,12	0,03	(-0,03 a	0,09)	1,35	(0,76 a	2,42)
Meningitis meningocócica	0,18	0,1	-0,07	(-0,14 a	0)	0,58	(0,35 a	0,97)
Meningitis por *Haemophilus influenzae*	0,06	0,09	0,03	(-0,02 a	0,08)	1,61	(0,79 a	3,27)
Leishmaniasis visceral	0	0,08	0,08	(0,05 a	0,12)	0	(0 a	0)
Mortalidad por malaria	0,01	0,05	0,03	(0 a	0,07)	3,43	(0,96 a	12,2)
Tuberculosis	0,03	0,02	-0,01	(-0,04 a	0,02)	0,79	(0,24 a	2,54)
Tétanos neonatal	0,01	0,01	0,01	(-0,01 a	0,02)	1,92	(0,23 a	16,1)
Rabia humana	0	0	0	(-0,01 a	0)	0	(0 a	0)

Fuente: datos del Sivigila

IRA: infección respiratoria aguda; ESAVI: eventos supuestamente atribuidos a la vacunación o inmunización; EDA: enfermedad diarreica aguda

* En rojo se identifica el régimen con la tasa más alta para cada evento y, en verde, la menor. No ví ningún color.

† Las tasas se ordenan de la más alta a la más baja para el régimen subsidiado.

‡ Las desigualdades absolutas negativas implican brechas que desfavorecen la población afiliada al régimen contributivo.

§ Las desigualdades relativas menores de uno implican brechas que desfavorecen la población afiliada al régimen contributivo.

Respecto a las desigualdades absolutas, se evidenció que la mayor brecha que desfavorece a la población afiliada al régimen subsidiado, se presentó para malaria por *P. falciparum* (82,31 casos adicionales por 100.000 afiliados), violencia de género (sic) (71,50 por 100.000 afiliados) y malaria por *P. vivax* (71,07 por 100.000 afiliados); en cuanto a las brechas relativas, también se presentó desigualdad para el régimen subsidiado, específicamente en los eventos de mortalidad por desnutrición en menores de cinco años (31,74 veces mayor el riesgo en el régimen subsidiado que en el contributivo), malaria por *P. falciparum*^12^ y malaria asociada a formas mixtas ^12^ (cuadro 1).

En el análisis por departamentos, se evidenció que dos de los tres eventos más frecuentes en el régimen contributivo (agresiones por animales potencialmente transmisores de rabia y varicela) presentaron tasas más altas en Bogotá y Antioquia. Por su parte, la violencia de género (sic) presentó las mayores tasas en el régimen subsidiado en los departamentos de Santander, Valle del Cauca, Huila y Nariño (cuadro 2).

**Cuadro 2:** Tasas de incidencia y mortalidad (ajustadas por edad y sexo) por 100.000 personas, desigualdades absoluta y relativa por régimen de afiliación y departamento para los cinco eventos de mayor frecuencia. Colombia, 2015

Agresiones por animales potencialmente transmisores de rabia				Varicela				Violencia de género (sic)				Dengue			Chikungunya			
Departamento	Contributivo	Subsidiado	Desigualdad absoluta‡ (IC95%)	Desigualdad relativa§ (IC95%)	Departamento	Contributivo	Subsidiado	Desigualdad absoluta‡ (IC95%)	Desigualdad relativa§ (IC95%)	Departamento	Contributivo	Subsidiado	Desigualdad absoluta‡ (IC95%)	Desigualdad relativa§ (IC95%)	Departamento	Contributivo	Subsidiado	Desigualdad absoluta‡ (IC95%)	Desigualdad relativa§ (IC95%)	Departamento	Contributivo	Subsidiado	Desigualdad absoluta‡ (IC95%)	Desigualdad relativa§ (IC95%)Desigualdad relativa§ (IC95%)
Antioquia	27,12	14,27	-12,85 (-15,13 a -10,57)	0,53 (0,47 a 0,6)	Bogotá	126,54	14,77	-111,78 -115,29 a -108,26)	0,12 (0,1 a 0,13)	Santander	9,45	22,54	13,09 (9,48 a 16,7)	2,39 (1,88 a 3,03)	Valle Del Cauca	73,22	15,16	-58,06 (-61,93 a -54,19)	0,21 (0,18 a 0,23)	Valle del Cauca	33,91	21,28	-12,63 (-20,12 a -5,14)	0,63 (0,49 a 0,8)
Valle Del Cauca	24,00	14,25	-9,75 (-12,35 a -7,14)	0,59 (0,51 a 0,69)	Atlántico	27,93	11,69	-16,25 (-19,9 a -12,59)	0,42 (0,35 a 0,51)	Antioquia	23,21	19,4	-3,81 (-6,18 a -1,44)	0,84 (0,75 a 0,94)	Tolima	23,79	14,39	-9,4 (-14,56 a -4,23)	0,6 (0,46 a 0,8)	Tolima	13,22	8,92	-4,3 (-13,4 a 4,79)	0,67 (0,32 a 1,42)
Cauca	6,17	12,82	6,65 (2,91 a 10,39)	2,08 (1,24 a 3,48)	Córdoba	5,42	10,82	5,4 (2,24 a 8,56)	2 (1,2 a 3,33)	Valle Del Cauca	16,96	18,37	1,41 (-1,14 a 3,96)	1,08 (0,94 a 1,25)	Santander	24,66	11,51	-13,15 (-16,87 a -9,42)	0,47 (0,37 a 0,6)	Meta	11,13	6,27	-4,86 (-15,07 a 5,34)	0,56 (0,21 a 2)
Bogotá	62,10	12,81	-49,3 (-52,07 a -46,52)	0,21 (0,18 a 0,24)	Valle Del Cauca	25,61	10,66	-14,95 (-17,46 a -12,43)	0,42 (0,35 a 0,49)	Huila	3,93	10,75	6,82 (3,62 a 10,02)	2,73 (1,51 a 4,96)	Huila	7,43	11,47	4,04 (0,19 a 7,9)	1,54 (0,98 a 2,44)	Casanare	5,29	4,99	-0,3 (-7,25 a 6,66)	0,94 (0,25 a 3,54)
Nariño	3,74	10,70	6,95 (3,95 a 9,96)	2,86 (1,5 a 5,45)	Antioquia	32,35	9,75	-22,6 (-24,85 a -20,36)	0,3 (0,26 a 0,35)	Nariño	1,90	10,68	8,78 (6,26 a 11,3)	5,62 (2,31 a 13,69)	Cesar	5,26	11,37	6,11 (2,65 a 9,57)	2,16 (1,27 a 3,68)	Quindío	3,01	4,73	1,72 (-0,9 a 4,33)	1,57 (0,8 a 3,09)
Cundinamarca	25,21	10,36	-14,85 (-18,41 a -11,3)	0,41 (0,33 a 0,52)	Nariño	2,81	7,45	4,64 (2,08 a 7,2)	2,65 (1,26 a 5,6)	Boyacá	3,94	8,42	4,49 (1,6 a 7,38)	2,14 (1,25 a 3,68)	Meta	14,62	10,06	-4,55 (-9,16 a 0,06)	0,69 (0,47 a 1)	Huila	3,68	4,44	0,77 (-0,99 a 2,52)	1,21 (0,79 a 1,86)
Boyacá	9,16	9,05	-0,12 (-3,77 a 3,54)	0,99 (0,66 a 1,47)	Sucre	3,54	6,97	3,44 (-0,05 a 6,92)	1,97 (0,82 a 4,76)	Cundinamarca	11,19	7,98	-3,21 (-5,86 a -0,56)	0,71 (0,54 a 0,95)	Antioquia	20,55	8,90	-11,65 (-13,56 a -9,75)	0,43 (0,37 a 0,5)	Antioquia	5,30	4,11	-1,19 (-2,49 a 0,12)	0,78 (0,58 a 1,03)
Atlántico	6,86	8,77	1,91 (-0,31 a 4,13)	1,28 (0,96 a 1,71)	Bolívar	7,90	6,77	-1,12 (-3,72 a 1,48)	0,86 (0,61 a 1,21)	Bolívar	1,99	7,11	5,12 (3,34 a 6,9)	3,57 (1,97 a 6,45)	N. de Santander	7,25	8,52	1,27 (-1,91 a 4,45)	1,18 (0,77 a 1,78)	Cundinamarca	7,46	3,54	-3,92 (-5,84 a -2)	0,47 (0,33 a 0,68)
Santander	10,43	8,19	-2,25 (-4,94 a 0,45)	0,78 (0,58 a 1,05)	N. de Santander	9,07	6,50	-2,57 (-5,87 a 0,74)	0,72 (0,48 a 1,08)	Casanare	2,06	6,76	4,7 (0,58 a 8,82)	3,28 (0,99 a 10,86)	Quindío	6,05	8,51	2,46 (-2,3 a 7,22)	1,41 (0,73 a 2,72)	Santander	4,72	2,53	-2,19 (-9,02 a 4,64)	0,54 (0,1 a 2,73)
N. de Santander	5,56	7,75	2,19 (-0,69 a 5,07)	1,39 (0,88 a 2,22)	Cundinamarca	26,80	6,48	-20,31 (-23,71 a -16,92)	0,24 (0,18 a 0,32)	N. de Santander	2,70	6,62	3,92 (1,63 a 6,21)	2,45 (1,31 a 4,6)	Bolívar	3,00	8,44	5,44 (3,4 a 7,47)	2,81 (1,72 a 4,59)	Córdoba	0,70	2,03	1,33 (0,34 a 2,33)	2,9 (1,06 a 7,95)
Tolima	6,80	7,73	0,94 (-2,15 a 4,02)	1,14 (0,74 a 1,75)	Boyacá	8,10	5,12	-2,98 (-6,16 a 0,21)	0,63 (0,39 a 1,01)	Cauca	1,40	6,59	5,19 (3,03 a 7,35)	4,7 (1,66 a 13,35)	Atlántico	9,09	8,27	-0,82 (-3,18 a 1,55)	0,91 (0,69 a 1,19)	Cauca	1,41	1,91	0,5 (-0,94 a 1,94)	1,36 (0,54 a 3,43)
Córdoba	2,01	7,16	5,15 (3,01 a 7,3)	3,57 (1,57 a 8,1)	Santander	16,09	4,76	-11,33 (-14,14 a -8,53)	0,3 (0,21 a 0,41)	Caldas	4,54	6,24	1,7 (-1,39 a 4,78)	1,37 (0,77 a 2,45)	Sucre	3,20	7,34	4,14 (0,75 a 7,52)	2,29 (0,91 a 5,75)	Magdalena	0,75	1,65	0,9 (-0,31 a 2,11)	2,21 (0,59 a 8,2)
Bolívar	2,39	6,46	4,07 (2,28 a 5,87)	2,7 (1,56 a 4,69)	Huila	3,81	4,41	0,6 (-2,02 a 3,22)	1,16 (0,6 a 2,23)	Bogotá	14,92	6,01	-8,91 (-10,56 a -7,26)	0,4 (0,32 a 0,51)	Cundinamarca	7,89	5,10	-2,79 (-4,97 a -0,61)	0,65 (0,45 a 0,92)	Arauca	0,38	1,59	1,21 (0,13 a 2,3)	4,2 (0,56 a 31,39)
Huila	3,98	6,20	2,22 (-0,61 a 5,04)	1,56 (0,84 a 2,9)	Tolima	6,11	3,69	-2,41 (-5,03 a 0,21)	0,6 (0,36 a 1,02)	Córdoba	1,19	5,67	4,48 (2,71 a 6,25)	4,77 (1,66 a 13,75)	Córdoba	2,26	4,22	1,96 (-0,06 a 3,98)	1,87 (0,84 a 4,13)	Caquetá	1,08	1,57	0,49 (-0,12 a 1,1)	1,45 (0,93 a 2,27)
Sucre	1,26	6,19	4,93 (2,47 a 7,39)	4,92 (1,17 a 20,72)	Magdalena	4,25	3,29	-0,96 (-3,41 a 1,49)	0,77 (0,42 a 1,43)	Sucre	0,71	5,21	4,5 (2,45 a 6,55)	7,31 (1,09 a 48,93)	Magdalena	1,84	3,85	2,01 (0,13 a 3,9)	2,1 (0,91 a 4,86)	Chocó	0,74	1,39	0,66 (-0,92 a 2,24)	1,89 (0,27 a 13,09)
Caldas	6,59	4,98	-1,6 (-4,8 a 1,6)	0,76 (0,43 a 1,32)	Caldas	6,36	3,24	-3,12 (-6,04 a -0,21)	0,51 (0,27 a 0,97)	Atlántico	3,51	5,19	1,68 (0,03 a 3,33)	1,48 (1 a 2,2)	Putumayo	0,72	3,59	2,88 (-0,52 a 6,28)	5,01 (0,15 a 200)	Atlántico	0,91	1,39	0,48 (-0,73 a 1,69)	1,52 (0,5 a 4,67)
Magdalena	3,41	4,98	1,57 (-0,82 a 3,97)	1,46 (0,77 a 2,76)	Arauca	0,84	2,56	1,72 (-1,81 a 5,25)	3,06 (0,11 a 88,57)	Cesar	1,50	5,14	3,64 (1,57 a 5,71)	3,42 (1,3 a 9,01)	Casanare	5,12	3,31	-1,81 (-6,08 a 2,46)	0,65 (0,24 a 2)	Bolívar	0,62	1	0,38 (-0,75 a 1,51)	1,61 (0,34 a 7,68)
Quindío	4,68	4,59	-0,09 (-3,89 a 3,7)	0,98 (0,43 a 2,22)	Meta	6,58	2,55	-4,03 (-6,85 a -1,21)	0,39 (0,2 a 0,76)	Risaralda	2,94	4,43	1,5 (-1,05 a 4,05)	1,51 (0,76 a 3,01)	Caldas	2,20	2,47	0,27 (-1,76 a 2,3)	1,12 (0,47 a 2,68)	N. de Santander	0,60	0,95	0,35 (-0,82 a 1,52)	1,58 (0,34 a 7,44)
Meta	5,05	4,42	-0,63 (-3,48 a 2,22)	0,87 (0,48 a 1,6)	Caquetá	1,55	2,43	0,88 (-2,51 a 4,27)	1,57 (0,21 a 11,58)	Meta	2,02	4,15	2,13 (-0,15 a 4,41)	2,05 (0,92 a 4,56)	Risaralda	3,35	2,32	-1,03 (-3,22 a 1,15)	0,69 (0,31 a 1,54)	Boyacá	1,44	0,77	-0,68 (-2,16 a 0,8)	0,53 (0,16 a 1,8)
Risaralda	6,00	4,39	-1,61 (-4,57 a 1,36)	0,73 (0,41 a 1,32)	Cesar	3,02	2,33	-0,7 (-2,92 a 1,52)	0,77 (0,35 a 1,69)	Quindío	1,7	3,42	1,73 (-1,1 a 4,55)	2,01 (0,63 a 6,46)	Boyacá	1,63	2,24	0,61 (-1,04 a 2,27)	1,38 (0,56 a 3,37)	Vichada	0,27	0,67	0,4 (-0,07 a 0,86)	2,48 (1,09 a 5,66)
Cesar	1,08	3,27	2,19 (0,49 a 3,89)	3,03 (0,96 a 9,59)	Cauca	2,08	2,26	0,18 (-1,79 a 2,16)	1,09 (0,43 a 2,77)	Caquetá	0,34	3,11	2,77 (0,34 a 5,19)	9,07 (0,16 a 500)	Arauca	0,73	2,17	1,44 (-1,83 a 4,72)	2,99 (0,08 a 100)	Putumayo	0,78	0,66	-0,12 (-5,01 a 4,77)	0,84 (0 a 600)
La Guajira	1,35	2,74	1,4 (-0,87 a 3,66)	2,04 (0,46 a 9,05)	Quindío	2,32	2,11	-0,21 (-2,83 a 2,41)	0,91 (0,28 a 2,98)	Tolima	1,64	3,05	1,41 (-0,3 a 3,12)	1,86 (0,82 a 4,2)	La Guajira	1,04	1,98	0,94 (-1,03 a 2,91)	1,9 (0,35 a 10,43)	Nariño	0,44	0,65	0,21 (-1,28 a 1,7)	1,47 (0,08 a 30)
Putumayo	0,94	2,38	1,44 (-1,97 a 4,85)	2,53 (0,11 a 56,57)	La Guajira	0,94	2,02	1,08 (-0,83 a 2,98)	2,15 (0,36 a 12,76)	La Guajira	0,52	2,93	2,41 (0,69 a 4,13)	5,64 (0,55 a 60)	Caquetá	1,24	1,98	0,73 (-2,31 a 3,77)	1,59 (0,17 a 14,88)	Cesar	0,96	0,64	-0,32 (-1,89 a 1,26)	0,67 (0,09 a 5)
Casanare	2,25	2,18	-0,07 (-3,12 a 2,98)	0,97 (0,25 a 3,83)	Risaralda	2,84	1,84	-1 (-2,99 a 0,99)	0,65 (0,27 a 1,58)	Magdalena	2,23	2,63	0,39 (-1,48 a 2,27)	1,18 (0,53 a 2,63)	Nariño	0,36	1,26	0,9 (-0,07 a 1,87)	3,49 (0,44 a 27,6)	Risaralda	0,59	0,61	0,02 (-0,91 a 0,96)	1,04 (0,22 a 4,96)
Caquetá	0,9	1,97	1,07 (-1,65 a 3,79)	2,19 (0,17 a 28,8)	Putumayo	0,33	0,95	0,62 (-1,45 a 2,69)	2,85 (0,02 a 500)	Putumayo	0,28	2,2	1,92 (-0,48 a 4,31)	7,78 (0,03 a 2000)	Cauca	1,3	1,21	-0,09 (-1,63 a 1,44)	0,93 (0,28 a 3,09)	Caldas	0,65	0,45	-0,19 (-1,17 a 0,78)	0,7 (0,12 a 4,18)
Arauca	0,56	1,72	1,16 (-1,73 a 4,05)	3,07 (0,05 a 187,4)	Casanare	1,82	0,91	-0,91 (-3,36 a 1,54)	0,5 (0,08 a 3,06)	Arauca	0,27	1,59	1,32 (-1,03 a 3,67)	5,91 (0,02 a 2000)	Chocó	0,32	0,81	0,5 (-1,36 a 2,35)	2,55 (0,01 a 456,79)	La Guajira	0,30	0,40	0,1 (-0,6 a 0,8)	1,35 (0,14 a 12,67)
Vaupés	0,1	0,5	0,4 (-4,01 a 4,81)	5,22 (0 a 90000000000000000	Amazonas	0,25	0,45	0,21 (-2,89 a 3,3)	1,83 (0 a 100000)	Vichada	0,09	0,78	0,69 (-2,32 a 3,71)	8,93 (0 a 500000000000)	Guaviare	0,39	0,56	0,17 (-3,52 a 3,85)	1,42 (0 a 8000)	Guaviare	0,34	0,40	0,06 (-3,41 a 3,54)	1,18 (0 a 10000)
Guaviare	0,18	0,48	0,3 (-2,48 a 3,09)	2,68 (0 a 600000)	Chocó	0,05	0,4	0,35 (-0,57 a 1,27)	7,67 (0 a 2000000)	Chocó	0,09	0,63	0,54 (-0,63 a 1,71)	7,05 (0 a 100000)	Amazonas	0,29	0,31	0,02 (-3,09 a 3,12)	1,06 (0 a 40000)	Sucre	0,21	0,26	0,05 (-0,79 a 0,89)	1,26 (0,03 a 61,78)
Amazonas	0,40	0,41	0 (-3,62 a 3,63)	1,01 (0 a 8000)	Vaupés	0,07	0,36	0,29 (-3,47 a 4,05)	5,19 (0 a 60000000000000000000	Amazonas	0,11	0,53	0,42 (-2,16 a 3)	4,74 (0 a 30000000)	Vichada	0,12	0,21	0,09 (-2,66 a 2,84)	1,75 (0 a 4000000000)	Guainía	0,16	0,18	0,02 (-3,86 a 3,89)	1,11 (0 a 30000000000)
Vichada	0,17	0,29	0,12 (-3,1 a 3,35)	1,75 (0 a 200000000)	Guaviare	0,21	0,24	0,03 (-2,57 a 2,63)	1,14 (0 a 200000)	Guaviare	0,10	0,4	0,3 (-1,95 a 2,55)	4,11 (0 a 70000000)	Guainía	0,10	0,21	0,11 (-3,1 a 3,33)	2,12 (0 a 20000000000000)	San Andrés	0,16	0,02	-0,13 (-2,19 a 1,92)	0,15 (0 a 300000000)
San Andrés	0,65	0,28	-0,37 (-3,9 a 3,16)	0,43 (0 a 7000)	Vichada	0,03	0,21	0,17 (-1,51 a 1,86)	6,62 (0 a 7000000000000000000)	Guainía	0,06	0,35	0,3 (-2,55 a 3,14)	6,33 (0 a 700000000000000000)	San Andrés	0,54	0,1	-0,44 (-3,16 a 2,27)	0,18 (0 a 1000000)	Amazonas	0,05	0,01	-0,04 (-1,74 a 1,66)	0,2 (0 a 20000000000000000)
Guainía	0,12	0,11	-0,01 (-3,4 a 3,38)	0,9 (0 a 900000000000)	Guainía	0,02	0,14	0,12 (-1,65 a 1,89)	7 (0 a 6E+28)	Vaupés	0,02	0,16	0,14 (-2,14 a 2,42)	7,3 (0 a 2E+34)	Vaupés	0,00	0,01	0,01 (-0,27 a 0,28)	0 (0 a 0)	Vaupés	0,01	0,01	0 (-1,08 a 1,08)	0,7 (0 a 2E+58)
Chocó	0,06	0,06	0 (-0,74 a 0,73)	0,93 (0 a 200000)	San Andrés	0,6	0,10	-0,5 (-3,33 a 2,33)	0,17 (0 a 800000)	San Andrés	0,16	0,04	-0,12 (-1,68 a 1,44)	0,24 (0 a 20000000000)	Bogotá	0,00	0,00	0 (0 a 0)	0 (0 a 0)	Bogotá	0,00	0,00	0,00 0 (-0,03 a 0,04) 0	(0 a 0)

Para otros eventos de escaso reporte, en términos absolutos, como accidente ofídico, leishmaniasis, lepra, tuberculosis (pulmonar y fármacorresistente), mortalidad perinatal, morbilidad materna extrema y mortalidad materna, se reportaron mayores tasas de incidencia en el régimen subsidiado en todos los departamentos. Además, hubo eventos de notificación obligatoria que, en la mayoría de los departamentos, solo se presentaron en el régimen subsidiado, como aquellos que afectan a los recién nacidos (sífilis congénita y gestacional).

## Discusión

En Colombia, existen desigualdades relacionadas con el régimen de afiliación al sistema de salud. A nivel nacional, las tasas de incidencia y mortalidad, ajustadas por edad y sexo, fueron más altas para el régimen subsidiado, en 37 eventos de notificación obligatoria al Sivigila, especialmente, aquellos relacionados con salud sexual y reproductiva, los que afectan a la población infantil y las enfermedades infecciosas desatendidas.

En el análisis por departamentos, se presentó mayor desigualdad que desfavorece a la población afiliada a este mismo régimen en casi todos los departamentos, principalmente, para los eventos de mortalidad por infección respiratoria aguda, mortalidad por enfermedad diarreica aguda, mortalidad materna, sífilis gestacional y congénita, violencia de género (sic), desnutrición, leishmaniasis, enfermedad de Chagas, malaria, lepra y tuberculosis; el evento de malaria por *P. falciparum* fue el que mayor desigualdad absoluta y relativa indicó.

La información obtenida en múltiples estudios alrededor del mundo, demuestra que el componente socioeconómico evaluado mediante diferentes variables representativas es un factor predisponente de mayor morbilidad y mortalidad prematura ^11^^-^^14^. En Colombia, la afiliación al régimen subsidiado tiene ciertas reglas de elegibilidad, con las cuales se da prioridad a poblaciones vulnerables y en condiciones socioeconómicas precarias ^15^. Estas condiciones de pobreza podrían desempeñar un papel fundamental en las altas tasas de notificación para algunos eventos en este subgrupo de población, en comparación con su contraparte afiliada al régimen contributivo.

No obstante, es pertinente aclarar que, de acuerdo con el marco conceptual de producción de inequidades propuesto por la OMS, el sistema de salud puede considerarse como un factor determinante social de la salud en sí mismo, que interacciona y puede modificar el efecto de los otros determinantes sociales. De tal manera, el sistema de salud está llamado a cumplir un papel activo en la reducción de las inequidades, no sólo mediante el acceso equitativo a los servicios de atención (servicios personales), sino también, en la planificación y ejecución de los programas de salud pública (servicios no personales). Por lo tanto en este caso, si bien los resultados se pueden explicar por los factores sociales, también el sistema de salud juega un papel fundamental ^16^.

Las condiciones de pobreza y vulnerabilidad en las que se encuentra la población afiliada al régimen subsidiado, se pueden asociar con diferentes barreras de acceso a los servicios básicos y de salud. Estas pueden ser barreras económicas, entre las que se encuentran costos de transporte, medicamentos y exámenes, o barreras geográficas, las cuales ocasionan que tengan menos facilidades para obtener atención, debido a su mayor dispersión ^16^. Estas barreras, finalmente, afectan la oportunidad de atención y la capacidad resolutiva de sus necesidades, lo cual produce un círculo vicioso entre mala salud y pobreza ^17^.

A pesar de la unificación del Plan Obligatorio de Salud (POS), existe una atención diferencial entre los dos regímenes de afiliación, llamada barrera organizativa, la cual es un factor que obstaculiza el contacto inicial con los servicios de salud (obstáculos de entrada) y, también, la atención oportuna después de que el paciente ingresa al centro de salud (obstáculos en el interior) ^18^. Aunque este estudio no puede corroborarlo, dicha barrera podría estar impactando negativamente el acceso a diferentes servicios de atención médica.

Otros estudios han evidenciado que la variable de aseguramiento al régimen subsidiado se asocia con: un menor acceso a todos los servicios de salud en general ^4^; menor utilización de servicios preventivos, de atención primaria y consulta especializada ^15^^,^^19^; mayor tiempo de espera para la asignación de citas de consulta general ^20^; paquetes de salud más limitados ^21^^,^^22^ y, específicamente para eventos maternos, menores tasas de atención y menos controles prenatales y posnatales ^23^^-^^25^.

Aunque esta investigación no hizo parte de un análisis de calidad de atención entre regímenes, la evidencia proporcionada ayuda a engrosar la documentación sobre las desigualdades en salud en el país.

El volumen de producción científica al respecto ha aumentado en la última década ^26^. Sin embargo, aún es escaso respecto a la medición de variables de aseguramiento, por lo que el presente estudio es un aporte para llenar dicho vacío de conocimiento. Estos resultados tienen impacto potencial en la salud pública, al demostrar la existencia de desigualdades en salud mediadas por la afiliación al régimen de salud, con peores resultados en la población del régimen subsidiado, hallazgo sobre el cual se puede lograr la toma de decisiones de política pública, encaminada a disminuir la estratificación social, que conlleve reducción de la exposición y la vulnerabilidad diferencial, de las consecuencias diferenciales o de ambas; además, pueden eliminar posibles barreras organizativas, y mejorar la calidad de la atención y las acciones de prevención y promoción.

De igual forma, este trabajo resulta un ejercicio ejemplar de la utilización y unificación de diferentes fuentes de información, entre ellas el Sivigila, la base de datos única de afiliados del Fosyga y las proyecciones poblacionales del Departamento Administrativo Nacional de Estadística (DANE), con las que no se habían realizado análisis de desigualdades hasta ahora. Es una prueba de la importancia y utilidad de los sistemas de información disponibles en el país y la necesidad de fortalecerlos para generar información para la acción.

Este análisis tiene limitaciones. Primero, si bien el Sivigila es la fuente oficial de información de vigilancia epidemiológica del país, la recolección y la depuración de los datos en ciertos lugares del país no se hacen de manera sistematizada, lo que afecta su calidad y la oportunidad de entregar la información para análisis como el presentado. Sin embargo, el Instituto Nacional de Salud ha venido mejorando la calidad y la oportunidad de dicha información ^26^.

Cabe anotar que, en el presente estudio, no se consideró una corrección del subregistro. Segundo, no fue posible la desagregación a nivel municipal de las variables de afiliación al sistema de salud, por lo que no se pudieron obtener datos más precisos de las brechas existentes entre los regímenes contributivo y subsidiado, aunque la evaluación de las desigualdades a escala departamental brinda información valiosa a los tomadores locales de decisiones, dando cuenta de las desigualdades territoriales que persisten en nuestro país. Tercero, se podrían haber implementado métodos más complejos de evaluación de desigualdades, que consideraran el peso poblacional que incluyeran el ajuste por otras variables de condiciones socioeconómicas en modelos multivariados. No obstante, se utilizaron los métodos usuales para evaluar las desigualdades sociales, los cuales se consideran buenas aproximaciones iniciales de valoración de dicha desigualdad. Cuarto, para las enfermedades transmitidas por vectores, se utilizó el denominador de población general, ya que el país no cuenta con cifras sobre población en riesgo discriminada según el régimen de afiliación al sistema.

En Colombia, se evidencian desigualdades sociales en salud que afectan la población afiliada al régimen subsidiado, una población que se ha caracterizado por pobres condiciones socioeconómicas y que requiere de una focalización de las intervenciones para lograr la disminución de estas diferencias, especialmente, aquellas que se consideran prevenibles, injustas e innecesarias.
